# DNA Methylation in the Ovary and Uterus of Mammalian Animal Models: Implications for Reproductive Function

**DOI:** 10.3390/genes17020228

**Published:** 2026-02-11

**Authors:** Oliwia Serej, Magdalena K. Kowalik, Robert Rekawiecki

**Affiliations:** InLife Institute of Animal Reproduction and Food Research, Polish Academy of Sciences, Trylińskiego 18 Str., 10-683 Olsztyn, Poland; o.serej@pan.olsztyn.pl

**Keywords:** methylation, pregnancy, uterus, ovaries, methyltransferases

## Abstract

DNA methylation is a key epigenetic modification that regulates gene expression and maintains genome stability, particularly in mammalian reproductive tissues. This review summarizes the current knowledge of DNA methylation and demethylation fluctuations with a specific focus on the regulation of ovarian development and uterine function during pregnancy. This modification primarily occurs at CpG-rich regions and is catalyzed by DNA methyltransferases (DNMTs): DNMT1 maintains existing patterns during replication, while DNMT3A and DNMT3B establish *de novo* methylation. Demethylation is mediated by ten-eleven translocation enzymes (TET1, TET2, and TET3), which oxidize 5-methylcytosine, ultimately replacing it with unmethylated cytosine. These processes play essential roles in folliculogenesis, oocyte maturation, steroidogenesis, and tissue-specific gene regulation. Understanding these epigenetic mechanisms provides important insights into veterinary medicine and offers potential applications in fertility preservation across diverse mammalian species. Consequently, further research is essential to elucidate the clinical implications of these epigenetic processes for improving reproductive health outcomes in animals.

## 1. Introduction

Epigenetics, particularly DNA methylation and demethylation, is one of the most important mechanisms regulating gene expression in mammalian tissues, without changing the DNA sequence itself. Epigenetic modifications enable cells to quickly respond to developmental and physiological signals. These processes are especially crucial in mammalian reproduction, as they control ovarian follicle growth, oocyte maturation, steroid hormone production, uterine remodeling, and maintenance of pregnancy. Most research investigating DNA methylation in the ovaries [1] and uterus [2] has focused on humans, mainly in relation to endometriosis [3,4], polycystic ovary syndrome (PCOS) [5,6], endometrial cancer [7], or infertility [8]. Publications concerning other mammalian species are considerably fewer. This gap is significant because animal models are essential for understanding basic reproductive physiology, interspecies differences, and the practical potential of this knowledge in veterinary medicine. In this review, we have collected and discussed the current state of knowledge on the roles of DNA methylation and demethylation in the ovaries and uteri of mammalian animal models (excluding humans). Particular attention is given to dynamic methylation changes during key reproductive processes: folliculogenesis, steroidogenesis, corpus luteum function, and endometrial remodeling in early pregnancy. Our goal is to demonstrate how important these epigenetic mechanisms are for reproductive health and fertility in mammals, including from a veterinary perspective.

## 2. The Role of DNA Methylation in Biological Processes

DNA methylation is an enzymatic modification process that plays a pivotal role in regulating gene function and maintaining genome stability. It is classified as an epigenetic modification, meaning it does not alter the DNA sequence itself [9]. The mechanism of the methylation reaction involves the transfer of the -CH3 methyl group from S-adenosylmethionine (SAM) to the fifth cytosine carbon atom, forming 5-methylcytosine (5mC). DNA methylation occurs predominantly on CpG (cytosine–guanine) islands, which are highly conserved regions, as is the case in mice [10].

### 2.1. CpG Islands

CpG islands are DNA regions with a high density of cytosine–guanine dinucleotides, typically defined as sequences longer than 500 base pairs with a GC content exceeding 50%. In mammals, CpG islands are found in approximately 60–70% of genes in the promoter regions [11]. These regions are typically hypomethylated under normal physiological conditions, enabling transcriptional activation at gene promoters. Unmethylated CpG islands exhibit an open chromatin structure that facilitates transcription factor and RNA polymerase binding, enabling active gene expression [12]. During regulatory processes, gene expression can be silenced through hypermethylation, which increases CpG island methylation in promoters, thereby inhibiting transcription. CpG island methylation plays a crucial role in various biological processes in the reproductive system [10]. Abnormal methylation patterns in CpG islands can silence genes involved in germ cell development, preventing egg, embryo [13], and sperm formation [14]. Imprinting gene methylation can also lead to developmental defects and reproductive system malignancies, such as ovarian [15] and mammary gland [16] cancers.

### 2.2. Methyltransferases in the DNA Methylation Pathway

DNA methyltransferase (DNMT) enzymes are responsible for DNA methylation. To date, five DNMTs have been identified in mammals: DNMT1, DNMT2, DNMT3A, DNMT3B, and DNMT3l [17] (Table 1). Each is involved in maintaining methylation or establishing it *de novo*. DNMT1 exhibits its highest activity during cell division, thereby transmitting the methylation pattern to daughter cells [18]. This process involves the recruitment of DNMT1 by ubiquitin-like proteins containing PHD and RING finger domains (1UHRF1), which recognize hemimethylated DNA sequences formed after cell division [19]. DNMT1 transfers a methyl group to the new DNA strand, maintaining the parent strand’s methylation pattern. This process is critical for maintaining appropriate methylation levels in cells after mitotic and meiotic divisions, as such methylation is essential for proper gene expression regulation. DNMT1 is the most structurally complex enzyme in the methyltransferase family [20]. It contains a CXXC domain with a conserved sequence of two cysteine pairs that binds unmethylated DNA, a PCNA-binding domain at the N-terminus that synchronizes DNA methylation with replication, and a catalytic domain responsible for transferring SAM to cytosine [21].

DNMT3A and DNMT3B are responsible for *de novo* methylation. Both possess a PWWP domain (a region that directs enzyme activity to specific methylation sites) containing a conserved Pro-Trp-Trp-Pro amino acid sequence responsible for directing enzyme activity to a specific methylation site. In addition, they contain an ADD domain, which enables selective methylation, and a catalytic domain responsible for methyl group transfer [22]. The specificity of DNMT3A and DNMT3B lies in their ability to catalyze the addition of a methyl group to regions that have not previously been methylated. The presence of these enzymes is crucial during embryonic development and cell differentiation, when newly established methylation patterns are established. DNMT3A primarily exhibits the highest activity in gene promoters [23]. DNMT3B acts similarly to DNMT3A but with a different target; it methylates regions characterized by tandem CpG repeats, such as centromeres and telomeres, which are crucial for epigenetic regulation in embryonic and somatic cell development [22].

DNMT3l lacks catalytic activity but enhances the efficiency of DNMT3A and DNMT3B through its ADD domain, which stabilizes their activity and prevents ubiquitination and proteasomal degradation [24]. DNMT3l has been shown to function as a homodimer or in complex with DNMT3A, allowing methylation of two closely spaced CpG islands. This complex comprises two DNMT3l and two DNMT3A proteins, which form a tetramer. This process involves the interaction of regulatory domains (RDs) located at the C-termini of DNMT3A and DNMT3l, which enter the complex. Because of the functions described above, the presence of DNMT3l is especially important during embryogenesis, when new methylation patterns are established *de novo* [25].

The final member of the methyltransferase family, DNMT2, is less active than the other enzymes in this group. It possesses a catalytic domain that is common in DNA methyltransferases; however, it exhibits low DNA catalytic activity. DNMT2 primarily methylates tRNA, specifically adding a methyl group to the cytosine at position 38, which stabilizes the tRNA’s secondary structure and makes it harder for ribonucleases to degrade. DNMT2 helps regulate translation and protect tRNA. Its function is evolutionarily conserved across all organisms, from yeast to mammals. DNMT2 mutations or changes in activity can lead to cellular changes, such as altered stress responses and transcriptome stability [26].

### 2.3. Demethylation as a Process of Controlling DNA Methylation

**Figure 1:**
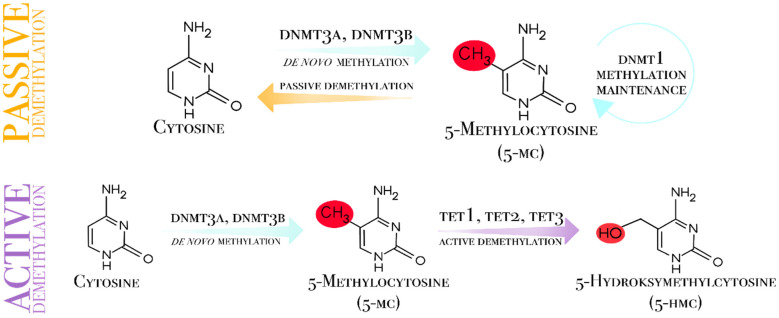
Mechanism of DNA methylation and demethylation. DNMT3A and DNMT3B exhibit high affinity for unmethylated DNA and catalyze de novo methylation in CpG islands. DNMT1 restores the methylation pattern during cell replication on the hemimethylated DNA strand. The demethylation process can be divided into two groups: passive and active. Passive demethylation involves the loss of a methyl group as a result of cell division. In active demethylation, TET enzymes play a central role by oxidizing 5-methylcytosine to 5-hydroxymethylcytosine; through further conversions, this process can ultimately restore the unmethylated state.

Demethylation, which eliminates methyl groups from cytosine nucleotides, is a reversible DNA methylation process [27]. Demethylation can be passive or active. When methylation patterns are not restored during replication, passive demethylation occurs, leading to a progressive loss of methylation [18]. Active demethylation, an enzymatic process independent of replication, is catalyzed by the ten-eleven translocation (TET) family of enzymes (Figure 1).

### 2.4. The Role of Dioxygenases in the Course of DNA Demethylation

The TET protein family comprises three enzymes, TET1, TET2, and TET3, which are dioxygenases that require Fe^2+^ and α-ketoglutarate (2OG) [28]. The C-terminal catalytic domain of TET enzymes contains a double β-helix for Fe^2+^ binding and a cysteine-rich region. Additionally, TET1 and TET3 possess a zinc finger-shaped N-terminal CXXC domain that binds CpG motifs [29]. TET2 lost its CXXC domain through chromosomal recombination during evolution. This domain was transferred to a separate gene, CXXC4 (IDAX). The protein encoded by IDAX interacts functionally with TET2 and regulates its activity, although it is not a direct structural component of TET2 [30]. These enzymes use 2OG, oxygen, and Fe^2+^ ions to oxidize 5-methylcytosine (5mC) to 5-hydroxymethylcytosine (5hmC). TET enzymes can then further oxidize 5hmC to 5-formylcytosine (5fC). In the third step, TET converts 5fC to 5-carboxycytosine (5caC). The resulting 5fC and 5caC are recognized by the glycosidase TDG, removed from DNA, and replaced with unmethylated cytosine via base excision repair (BER), leading to demethylation [31]. TET1 mainly acts on gene promoters enriched in CpG islands, preventing hypermethylation-induced gene silencing, especially of genes that maintain cell pluripotency, such as *Nanog*, *Oct4*, and *Sox2* [32].

## 3. The Role of DNA Methylation in Ovarian Function

DNA methylation is a ubiquitous process in living organisms, with tissue-specific roles. In the ovaries, which undergo dynamic changes during the reproductive cycle, DNA methylation regulates genes involved in follicle maturation [33], steroid hormone action [34], secretion, and ovarian reserve maintenance [35]. Therefore, this process can help regulate ovarian function during the estrous cycle.

### 3.1. The Role of DNA Methylation and Demethylation in Oocyte and Ovarian Follicle Maturation

**Figure 2:**
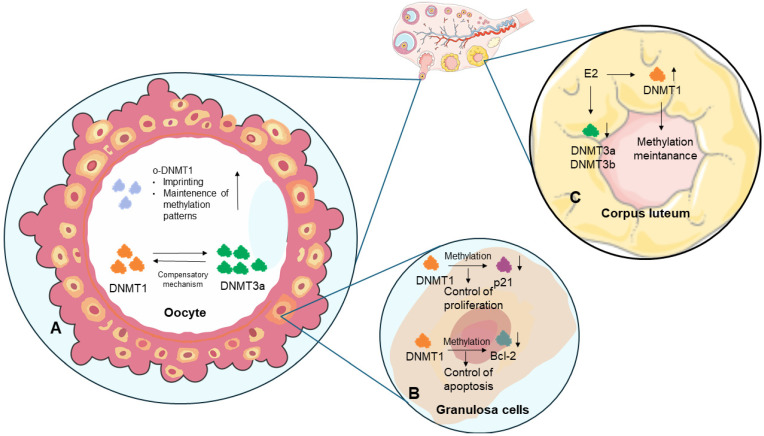
The role of DNA methylation in the oocyte and corpus luteum in mammals. (**A**) Oocyte-specific DNMT1 (o-DNMT1) accumulates during oocyte formation and is responsible for imprinting and maintaining methylation patterns (up arrow). In addition, there is a dependence in which the expression level of *DNMT1* is linked to the expression level of *DNMT3A* (a compensatory mechanism). (**B**) During granulosa cell development, DNMT1 catalyzes methylation of the *CDKN1A* promoter, which encodes the p21 protein, a tumor suppressor that inhibits cell proliferation. Reduced *CDKN1A* expression leads to decreased p21 levels (down arrow). DNMT1 also methylates the *Bcl-2* gene, which encodes the anti-apoptotic Bcl-2 protein, thereby reducing its abundance (down arrow). Both of these mechanisms help maintain the balance between proliferation and apoptosis in granulosa cells. (**C**) Estradiol (E2) acts on methyltransferases in the corpus luteum (CL), causing an increase in DNMT1 gene expression (up arrow) while simultaneously decreasing *DNMT3A* and *DNMT3B* mRNA levels (down arrow). This results in the maintenance of existing methylation patterns rather than the establishment of new ones. Image(s) provided by Servier Medical Art (https://smart.servier.com), licensed under CC BY 4.0 (https://creativecommons.org/licenses/by/4.0/ accessed on 10 December 2025).

Folliculogenesis is the maturation of ovarian follicles, which determines fertility and the proper development of the oocyte. The multi-stage process of folliculogenesis involves the growth, differentiation, and release of mature oocytes during ovulation. DNA methylation levels in oocytes have been found to change at each stage of meiosis and increase as oocytes mature [36]. Other studies have indicated that chromatin condensation in the oocyte is accompanied by a gradual increase in global DNA methylation, particularly during the transition from GV0 to GV1, which is associated with transcriptional silencing and the attainment of meiotic maturity [37]. Researchers have observed DNMT1 in cow oocytes during the late stages of oocyte differentiation and have demonstrated that its distribution changes during the early stages of embryo development, hypothesizing that DNMT1 in the nucleus at the 8–16 cell stage may help keep the methylation patterns of imprinted genes, as it does in mice. Thus, proper DNMT1 function is necessary for epigenetic imprinting in oocytes and early embryos [37]. It has been demonstrated that the oocyte-specific form of DNMT1 (oocyte-specific DNMT1; o-DNMT1) accumulates during oocyte formation [37]; furthermore, o-DNMT1 is actively involved in imprinting, or the maintenance of methylation patterns, in oocytes (Figure 2). Lack of o-DNMT1 activity impairs or eliminates imprinting, leading to the abnormal expression of genes involved in subsequent oocyte development [38]. Together, these findings suggest an association between progressive DNA methylation, chromatin remodeling, and the acquisition of oocyte developmental competence, despite differences in DNMT1 localization and dynamics.

During follicle growth, from the primary stage, the granulosa layer forms around the oocyte, supporting its development by providing nutrients. DNMT1 has been shown to methylate genes involved in cell cycle progression, e.g., *CDKN1A* (which encodes the tumor suppressor protein p21), resulting in reduced granulosa cell proliferation [39]. During cellular homeostasis, DNMT1 maintains appropriate methylation patterns in the *CDKN1A* promoter, ensuring normal granulosa cell proliferation. Reduced DNMT1 activity leads to *CDKN1A* overexpression, decreasing granulosa cell division and potentially inhibiting follicle maturation, which may contribute to conditions such as PCOS [40]. DNMT1 is crucial for regulating granulosa cell survival by modulating the apoptotic process. The *Bcl2* gene, which encodes a protein with anti-apoptotic properties, possesses promoter sequences that are methylated by DNMT1. By regulating DNA methylation, DNMT1 influences the balance between apoptosis and granulosa cell proliferation, which is important for the proper growth and development of ovarian follicles (Figure 2) [39].

DNMT1 is not the only methyltransferase involved in ovarian follicle maturation. DNMT3A and DNMT3B can also affect genes associated with ovarian follicle and oocyte maturation. *DNMT3B* expression increases with oocyte meiotic divisions, whereas *DNMT3A* expression is less pronounced, suggesting a role in *de novo* imprinting [41]. A compensatory mechanism has been demonstrated between DNMT1 and DNMT3A (Figure 2). This process involves a decrease in *DNMT1* expression, leading to an increase in *DNMT3A* mRNA expression; conversely, silencing *DNMT3A* results in an increase in *DNMT1* mRNA expression. Importantly, silencing *DNMT3A* and *DNMT1* does not increase *DNMT3B* expression [42]. It has been shown that silencing of *DNMT* family genes can lead to infertility, inhibition of meiotic divisions, or imprinting disorders [23], indicating that these enzymes are dependent on each other despite their different pathways of action.

Demethylation in oocytes occurs before ovulation in regions critical for activating imprinting-related genes. The mature oocyte has been shown to contain differentially methylated regions (DMRs) that undergo demethylation until implantation [43].

The promoter regions of genes involved in meiosis and oocyte development must undergo demethylation to be expressed. Two enzymes responsible for active DNA demethylation, TET1 and TET2, play key roles in ovarian follicle maturation and oogenesis; however, their functions are distinct. The TET1 enzyme primarily regulates chromosome segregation during meiotic divisions in maturing mouse oocytes. Studies involving *TET1* gene silencing have shown that loss of TET1 activity leads to abnormal chromosome segregation, DNA repair issues, and a reduced number of oocytes [44]. In contrast, although silencing the *TET2* gene does not affect oocyte number, it results in a noticeable delay in meiotic divisions. Additionally, the absence of TET2 activity results in a significant reduction in 5hmC levels in oocytes, thereby decreasing their quality [45].

TET1 actively participates in ovarian follicle maturation by regulating the expression of gonadotropic hormones, such as LH and FSH, by demethylating the promoters of their genes. These hormones are essential in the maturation of ovarian follicles [46]. Furthermore, TET1 influences the overall ovarian follicle reserve and oocyte quality. Studies have shown that *TET1* gene silencing reduces the number of ovarian follicles early in the development of a given organism, followed by their disappearance with age. Furthermore, *TET1* gene silencing dysregulates autophagy and oocyte ubiquitination, which may lead to premature oocyte aging and reduced fertilization capacity [47]. The TET3 enzyme is also involved in oocyte maturation. *TET3* silencing in oocytes has been demonstrated to result in a diminished or lost capacity to transition to metaphase II, which is necessary for fertilization [48]. In addition, a correlation has been established between the absence of TET3 activity and elevated methylation levels in the promoter regions of genes responsible for oocyte pluripotency, including *OCT4* and *NANOG*, which determine the developmental potential of the oocyte [45].

The above data indicate that DNA methylation and demethylation processes do not act independently but form a dynamically regulated feedback system during oocyte and follicle maturation. High activity of maintenance methylation (mainly o-DNMT1) in the final stages of oocyte maturation correlates with the need to preserve genomic imprinting [37,39], whereas the simultaneous increase in expression and activity of TET enzymes (particularly TET1 and TET3) enables the activation of key genes associated with meiosis and pluripotency (including *OCT4* and *NANOG*) [44,45,48]. Disruption of this balance in either direction, whether that is excessive methylation or insufficient demethylation, leads to similar reproductive phenotypes: inhibition of meiotic maturation, decreased oocyte quality, and accelerated depletion of the follicular reserve [42,44,47].

### 3.2. DNA Methylation in the Regulation of Steroidogenesis in Ovarian Follicles

DNA methylation has also been shown to regulate steroidogenesis in ovarian follicles. It primarily modulates gene expression in granulosa and theca cells, which are responsible for the biosynthesis of steroid hormones essential for follicular development and oocyte maturation. The *CYP19* gene, which encodes aromatase, an enzyme necessary for estrogen production, is hypomethylated in ovarian follicles, thereby enabling high aromatase expression and elevated estrogen secretion [49].

Researchers have shown that DNA methylation and the expression levels of DNA methyltransferases (*DNMT1*, *DNMT3A*, and *DNMT3l*) decrease with age in mouse ovaries. These epigenetic changes are linked to lower estrogen levels, which makes pregnancy difficult. However, the increase in *DNMT3B* expression during early sexual maturity and later aging stages does not offset the overall decline in DNA methylation. Disruptions in the expression of genes linked to estrogen synthesis can lead to reduced estrogen production and age-related infertility, which can occur when DNMTs are less active and methylation function is disrupted [42,50].

It has been demonstrated that the expression of *DNMT3A* and *DNMT3B* genes in the hypothalamus is subject to estrous cycle rhythms and the action of ovarian steroids. Administration of estradiol and P4 has been shown to increase the number of cells expressing *DNMT3A* and increase *DNMT3B* expression in hypothalamic paraventricular nuclei [51].

TET demethylation of the promoters of genes encoding enzymes important for steroid hormone production, such as *StAR CYP11A1*, and *3β-HSD*, increases their expression, thereby elevating follicular steroid levels. As follicles grow, the patterns of DNA methylation at these promoters change. For example, in studies of chickens, important steroidogenesis genes have been found to exhibit low methylation levels during steroidogenesis (e.g., in pre-ovulatory follicles), supporting estrogen and P4 synthesis. After ovulation (post-ovulatory follicles), increased methylation leads to gene silencing and reduced steroid production [52].

The convergence of epigenetic patterns observed in steroidogenesis across different species and successive phases of the reproductive cycle suggests the existence of a universal epigenetic mechanism. Hypomethylation of promoters of key steroidogenic genes (*CYP19A1*, *StAR*, *CYP11A1*, and *HSD3B*) consistently occurs during periods of highest steroidogenic activity, both in pre-ovulatory follicles and in the early corpus luteum [49,52]. In turn, remethylation of these same regions after ovulation (particularly evident in the case of *CYP19A1*) correlates with aromatase expression silencing and a shift in the steroidogenic profile toward progesterone predominance [49,50]. These observations indicate that dynamic changes in methylation levels at steroidogenic gene promoters may represent a primary mechanism enabling rapid and directed responses to gonadotropic and local paracrine signals [42,51,52].

### 3.3. The Role of DNA Methylation in Corpus Luteum Function

The corpus luteum (CL) is an endocrine gland formed by luteinization of the follicle that is ruptured after ovulation. The main function of this gland is the secretion of P4, which is essential for preparing and maintaining embryo implantation and early pregnancy. This hormone acts via the genomic pathway through nuclear receptors (PGRs), which exist in two isoforms: A (PGRA) and B (PGRB) [53]. Both the expression level of individual isoforms and the hormone itself change during the estrous cycle in cows [54]. Expression of *PGR* mRNA is also regulated by many luteotropic and luteolytic factors, which translate into the effects of P4 in the CL [55]. DNA methylation of the *PGRA* and *PGRB* promoters may also indirectly influence *PGR* expression. Studies employing HpaII/Mspl restriction enzymes have revealed DNMT1 mRNA expression in the CL during the estrous cycle, along with higher *PGRA* promoter methylation compared with that of the *PGRB* promoter; however, methylation of the individual isoforms remained consistent throughout the cycle [34]. Changes in expression levels were also observed for the *DNMT1*, *DNMT3A*, and *DNMT3B* methyltransferase genes, and DNMT1 activity was found to increase during the estrous cycle. In addition, changes in the expression of *DNMT3A* and *DNMT3B* correlate with the level of P4 secreted by the CL (unpublished data). Thus, DNA methylation may depend on P4 synthesis. Furthermore, locally secreted hormones may regulate DNA methylation levels in the porcine CL. Estradiol has been shown to reduce DNMT3A and DNMT3B protein levels and increase DNMT1 protein expression. Therefore, estradiol may favor the maintenance of existing DNA methylation patterns by altering DNMT1 protein expression rather than promoting *de novo* methylation mediated by DNMT3 enzymes (Figure 2) [56]. In addition, it has been shown that exposing the CL to endocrine-active compounds, such as methoxychlor or antiestrogens, increases global DNA methylation. This hypermethylation was linked to changes in the expression of *StAR*, *CYP11A*, and *3β-HSD* that are involved in steroidogenesis, and lower P4 production. These findings show that these epigenetic changes can disrupt the normal physiology of the luteal phase, potentially leading to luteal insufficiency and subfertility. These endocrine-active compounds also altered *DNMT* expression, suggesting that their effects on DNA methylation are mediated by changes in DNMT activity [57]. Epigenetic modifications can also affect P4 secretion by inhibiting the expression of genes that regulate CL function. The luteinization process involves increased DNA methylation at the promoter of the inhibin alpha gene, mediated by DNMT3A. This enzyme is associated with the inhibin alpha promoter region, indicating that DNMT3A is essential for the repression of inhibin alpha expression in the murine corpus luteum [58]. A similar effect has been observed for the *CYP19* gene, which becomes hypermethylated with the onset of CL formation in buffalo ovary [49]. No existing studies have examined the influence of demethylation on CL function; however, in our research, we have shown the highest mRNA expression of *TET1* and *TET2* on days 6–16, and *TET3* at the end of the cycle. In contrast, TET protein activity was high on days 2–16 and decreased during the last phase of the cycle. The gene expression levels of all enzymes (excluding TET3) and TET activity showed a positive correlation with P4 levels (unpublished data). These initial observations suggest that, in addition to DNA methylation, DNA demethylation may also contribute to the regulation of corpus luteum function; however, this possibility remains to be further investigated.

An analysis of the available literature and our own preliminary data allows us to propose a model of cooperation between methylation and demethylation in CL function: high expression and activity of DNMT1 during the early and midphases of CL function (supported by estradiol action) promote epigenetic stabilization characteristic of the luteal phenotype [34,56]. At the same time, a marked increase in the expression and activity of TET1 and TET2 enzymes during days 6–16 of the cycle correlates with the need to maintain epigenetic plasticity, enabling responses to luteotropic signals and preparing for potential luteolytic changes (unpublished data). The decline in TET activity toward the end of the functional cycle coincides with the loss of epigenetic plasticity and preparation of the tissue for regression processes. This balance between maintenance methylation (DNMT1) and active demethylation (TET1/TET2) appears to constitute one of the key mechanisms regulating the lifespan and functional competence of the CL [34,56,57]. It should be noted that current evidence for active DNA demethylation in CL function is limited, and some proposed mechanisms remain hypothetical and require experimental validation.

## 4. The Role of DNA Methylation in Mammalian Uterine Function and the First Trimester of Pregnancy

As previously mentioned, DNA methylation affects the synthesis of estradiol and progesterone in the ovary. These hormones regulate processes related to implantation and cyclic changes in the endometrium, ensuring coordination between ovulation and the preparation of the endometrium to receive the embryo. In addition, methyltransferase family genes are expressed in the bovine endometrium, suggesting that DNA methylation influences uterine function in mammals [59].

### 4.1. Effects of DNA Methylation on Endometrial Development and Function

*DNMT1* and *DNMT3B* expression levels have been found to change in the early days of the estrous cycle in the bovine endometrium, whereas *DNMT3A* expression decreases significantly from day 3 to day 7. However, these expression changes did not translate into detectable changes in global DNA methylation [60]. It has also been suggested that a compensatory mechanism exists between endometrial and myometrial tissues that can regulate DNA methylation. When endometrial tissue was exposed to a very low-frequency electromagnetic field, *DNMT1* expression decreased, and *DNMT3A* expression increased. In contrast, the myometrium showed the opposite response, with *DNMT3A* levels decreasing and *DNMT1* expression increasing. These results suggest that the DNA methylation machinery is regulated differently across the layers of the uterus. This distinct modulation of DNA methyltransferases could lead to distinct patterns of epigenetic remodeling in the endometrium and myometrium, potentially altering cellular and, potentially, global uterine function [61].

Researchers have investigated the role of DNA methylation in embryo implantation. DNA methylation inhibitors such as 5′-azacytidine (azacitidine) and 5-aza-2′-deoxycytidine (decitabine) have been shown to block decidualization in mouse endometrial stromal cells. This effect disrupts the physiological remodeling of the endometrium necessary for successful embryo implantation, leading to implantation failure and early pregnancy loss. Researchers have found that applying these demethylating agents alters gene expression linked to endometrial differentiation, rendering cells less able to perform the functions required to create a receptive endometrium [62].

Overall, research has demonstrated that DNA methylation plays a key role in regulating endometrial function and uterine receptivity. The tissue-specific expression of DNMTs facilitates distinct epigenetic remodeling in the uterus, enabling appropriate responses to hormonal signals. Disruption of these mechanisms hinders decidualization and implantation.

### 4.2. Role of DNA Methylation in the First Trimester of Pregnancy

In mammalian reproduction, DNA methylation plays a key role in maintaining the normal course of pregnancy, especially in its early stages. The first trimester of pregnancy encompasses the critical stages of embryonic development, from fertilization through the blastocyst stage to implantation.

During mammalian gametogenesis, DNA methylation patterns undergo reprogramming. The parental methylation patterns are removed, and a new, sex-specific methylation pattern is subsequently imposed, a process referred to as imprinting. This process is regulated by DNMTs such as DNMT3A, DNMT3B, and the non-catalytic DNMT3l. DNA methylation is essential for imprinting, silencing germline-specific genes, and repressing transposable elements, thereby protecting the integrity of the genome during germ cell development [63]. Collectively, these processes establish a stable, sex-specific epigenetic program that safeguards genome integrity and enables proper germ cell development and subsequent embryonic competence.

**Figure 3:**
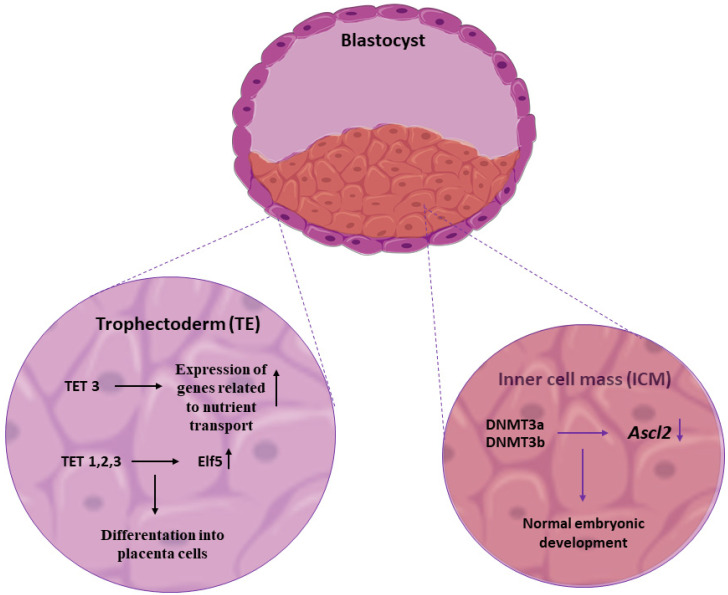
Role of methylation in the blastocyst. Two main cell lineages are formed after fertilization: the inner cell mass (ICM) and the trophectoderm (TE). DNMT3A and DNMT3B catalyze methylation of the *ASCL2* gene (down arrow) in the ICM, thereby inhibiting TE cell development. At the same time, TET dioxygenases in TE cells catalyze demethylation of the *Elf5* gene (up arrow), promoting TE cell differentiation. Image(s) provided by Servier Medical Art (https://smart.servier.com), licensed under CC BY 4.0 (https://creativecommons.org/licenses/by/4.0/ accessed on 10 December 2025).

Similar methylation-dependent regulation of trophoblast migration has been observed across porcine and bovine models, suggesting a conserved role for DNA methylation in placental morphogenesis. For example, methylation of regulatory regions in trophoblasts controls the expression of angiogenic and adhesive genes, such as *ANGPTL4* and Rap1, which are crucial for creating a functional placenta [64,65]. In pigs and cattle, methylation changes during pregnancy influence the expression of genes involved in the organization of the extracellular matrix (ECM), such as *COL1A2* and *COL7A1*, cytokine production, and angiogenesis pathways, such as *ANGPTL* and *RAP1* [66]. These genes are important for placental structure formation and nutrient exchange capacity. Decreased methylation of genes such as *ANGPTL4* and *RAP1* is associated with increased trophoblast migration, adhesion, and the development of placental structures that increase the surface area for nutrient exchange between the mother and fetus [67]. After fertilization, mammalian embryos develop into two main cell lineages: the inner cell mass (ICM) and the trophectoderm (TE). The ICM later forms the embryo proper, while the TE forms the placenta and other extraembryonic tissues. DNMTs such as DNMT3A and DNMT3B mediate methylation that silences trophoblast-specific genes, such as *ASCL2*, in the epiblast (the precursor of the ICM). Loss or inhibition of *DNMT3A* and *DNMT3B* expression leads to ectopic activation of *ASCL2* in epiblast cells, causing an abnormal cell-fate transition toward the trophectoderm lineage and disrupting normal embryonic development (Figure 3) [68]. Together, these findings position DNA methylation as a key integrator of trophoblast behavior, placental architecture, and embryonic lineage specification, linking epigenetic regulation directly to functional pregnancy outcomes.

In addition, the expression of TET enzymes (*TET1*, *TET2*, and *TET3*), which are involved in DNA demethylation, is highly variable in the placenta. These enzymes are detected in trophoblast progenitor cells, cytotrophoblasts, and syncytiotrophoblast cells. Studies in the mouse placenta have demonstrated that *TET1*, *TET2*, and *TET3* expression levels vary with differentiation stage and the specific placental microenvironment. Furthermore, *TET3* expression is significantly increased in differentiated trophoblast populations and correlates with the active transcription of genes involved in nutrient transport. This dynamic regulation of *TET* expression suggests their key role in epigenetic remodeling during trophoblast differentiation and adaptation of placental function [69].

*Elf5* is an important transcription factor that regulates trophoblast lineage differentiation. The *Elf5* promoter is heavily methylated and turned off in ICM cells. In the TE lineage, TET proteins demethylate the promoter, thereby activating *Elf5* expression (Figure 3). Thus, TET enzymes activate *Elf5* by demethylating it, thereby promoting trophoblast lineage specification and placental development [70].

Comparing epigenetic dynamics across the ovary–endometrium–placenta axis reveals several important convergences and functional oppositions. During the preimplantation phase, both granulosa cells and the endometrium have been found to exhibit elevated TET enzyme activity, which presumably facilitates the epigenetic plasticity required for rapid adaptation to fluctuating sex steroid levels [45,60,69]. Post-implantation, a distinct transition toward *de novo* methylation (DNMT3A/DNMT3B) is evident in the ICM lineage, whereas the trophoblast lineage sustains a comparatively elevated level of TET activity; this mechanism appears to be essential for cell fate determination and trophoblast specification [68,69,70]. Importantly, in both systems (granulosa cells and trophoblast), hypomethylation of the regulatory regions of genes associated with angiogenesis (e.g., *ANGPTL4*, *RAP1*) and adhesion strongly correlates with intensive vascular development and an increased maternal–fetal exchange surface area [64,65,66,67]. These observations suggest that, despite differences in the timing and extent of epigenetic changes, the entire female reproductive axis exhibits a common regulatory logic: high plasticity (dominance of demethylation) during developmental and adaptive phases, followed by stabilization of methylation patterns (predominance of DNMT1 and *de novo* methylation) during periods of maintenance of specialized functional phenotypes [63,68,70].

## 5. Research Limitations

Despite the growing number of studies indicating a significant role for DNA methylation in the regulation of ovarian and uterine function in animals, the current state of knowledge is constrained by several limitations that make the epigenetic mechanisms in reproductive physiology difficult to understand. Most available studies are restricted to model species such as mice and rats, as well as lower-degree livestock animals, including cattle, sheep, and pigs. Interspecies differences in the length and characteristics of the estrous cycle, endometrial structure, timing of implantation, and hormonal regulation may substantially influence DNA methylation patterns and their regulation within given species. Consequently, these factors significantly complicate, and in some cases preclude, direct comparisons of results obtained across different animal species. Many existing studies have relied on analyses of DNA methylation and demethylation levels or assessing changes in *DNMT* and *TET* gene expression in vitro; however, the findings are rarely supported by experimental manipulation of DNA methylation *in vivo*. Thus, it remains unclear whether methylation changes observed under laboratory conditions are also functionally relevant in living organisms. Although the present review primarily addresses the physiological regulation of DNA methylation in ovarian and uterine tissues in animal models, it should be noted that various environmental pollutants are increasingly implicated in disrupting these epigenetic processes, often with long-term consequences for female fertility. Well-known examples include transgenerational ovarian disease in rats after ancestral exposure to vinclozolin or methoxychlor [71], altered methylation of estrogen receptor promoters following developmental exposure to endocrine disruptors [72], and possible epigenetic alterations in the CL after neonatal exposure to endocrine-active compounds in gilts [57]. Very recent reports have also described the presence of microplastics in follicular fluid [73] and associations between PFAS exposure and reproductive parameters in mammals [74]. Clearly, more work is needed in relevant large-animal models to better understand how environmental factors interact with the female reproductive epigenome.

Given these limitations, the currently available data should be interpreted with caution. Furthermore, these limitations underscore the critical need for further research on the functional and temporal mechanisms of DNA methylation in animal reproductive tissues.

Future studies should focus on functional analyses of DNA methylation in the ovary and uterus using in vivo animal models and extend investigations beyond classical laboratory species. Particular emphasis should be placed on the temporal dynamics of epigenetic changes and their cell-type specificity. Such approaches will enable a more comprehensive understanding of the role of DNA methylation in regulating female reproductive function in animals.

## 6. Conclusions

DNA methylation is a key epigenetic mechanism that regulates gene expression and maintains genome stability in tissues, particularly in mammalian reproductive organs such as the ovaries, uterus, and placenta. This review demonstrates that this process, through the precise action of DNMTs (DNMT1, DNMT3A, DNMT3B, DNMT3l, DNMT2) and TET dioxygenases (TET1, TET2, TET3), significantly influences mechanisms in the mammalian female reproductive system, including oocyte and follicle maturation, steroidogenesis, CL function, endometrial development, and early pregnancy. Maintaining a balance between methylation and demethylation is essential for epigenetic homeostasis in reproductive cells. Disruptions in these mechanisms can lead to abnormal gene expression, fertility disorders, and issues with implantation and pregnancy maintenance. Although this review covers existing research on DNA methylation’s impact on steroidogenesis, some aspects—such as the role of demethylation in corpus luteum function—remain underexplored, and the contributions of individual methyltransferases and TET enzymes to uterine remodeling require further investigation. A deeper understanding of these relationships could provide deeper insights into fertility regulation and contribute to new diagnostic and therapeutic approaches for reproductive disorders.

## Figures and Tables

**Table 1:** Mechanism of action of the methyltransferase family.

Feature	DNMT1	DNMT2	DNMT3A	DNMT3B	DNMT3l
Function	Maintenance of methylation during replication	tRNA methylation	*de novo* methylation	*de novo* methylation	Enhances DNA methylation efficiency
Action	Recruitment by UHRF1 to hemimethylated DNA	Stabilizes tRNA secondary structure	Forms homodimers or complexes with DNMT3l	Methylates tandem CpG repeats. (centromeres, telomeres)	Forms a complex with DNMT3A

## Data Availability

No new data were created or analyzed in this study.
